# Supplementary material for: Peripheral NOD-like receptor deficient inflammatory macrophages trigger neutrophil infiltration into the brain disrupting daytime locomotion
Source: Commun Biol. 2022 May 16;5:464. doi: 10.1038/s42003-022-03410-z (PMC9110401; doi:10.1038/s42003-022-03410-z)
Supplement: Supplementary file 1 — Supplementary Information [file 42003_2022_3410_MOESM1_ESM.pdf]

Supplemental Information for

Peripheral NOD-like receptor deficient inflammatory macrophages trigger neutrophil infiltration into the brain disrupting daytime locomotion

Victoria Kwon[#], Peiwen Cai[#], Cameron T. Dixon[#], Victoria Hamlin, Caroline G. Spencer, Alison M. Rojas, Matthew Hamilton, and Celia E. Shiau[#§*]

Department of Biology, University of North Carolina at Chapel Hill, Chapel Hill, NC
[§] Department of Microbiology and Immunology, University of North Carolina at Chapel Hill, Chapel Hill, NC

[#] These authors contributed equally.
*Author for correspondence
Email: shiauce@unc.edu

This file contains:
Supplementary Figures and Legends 1-11

Supplementary Figure 1.

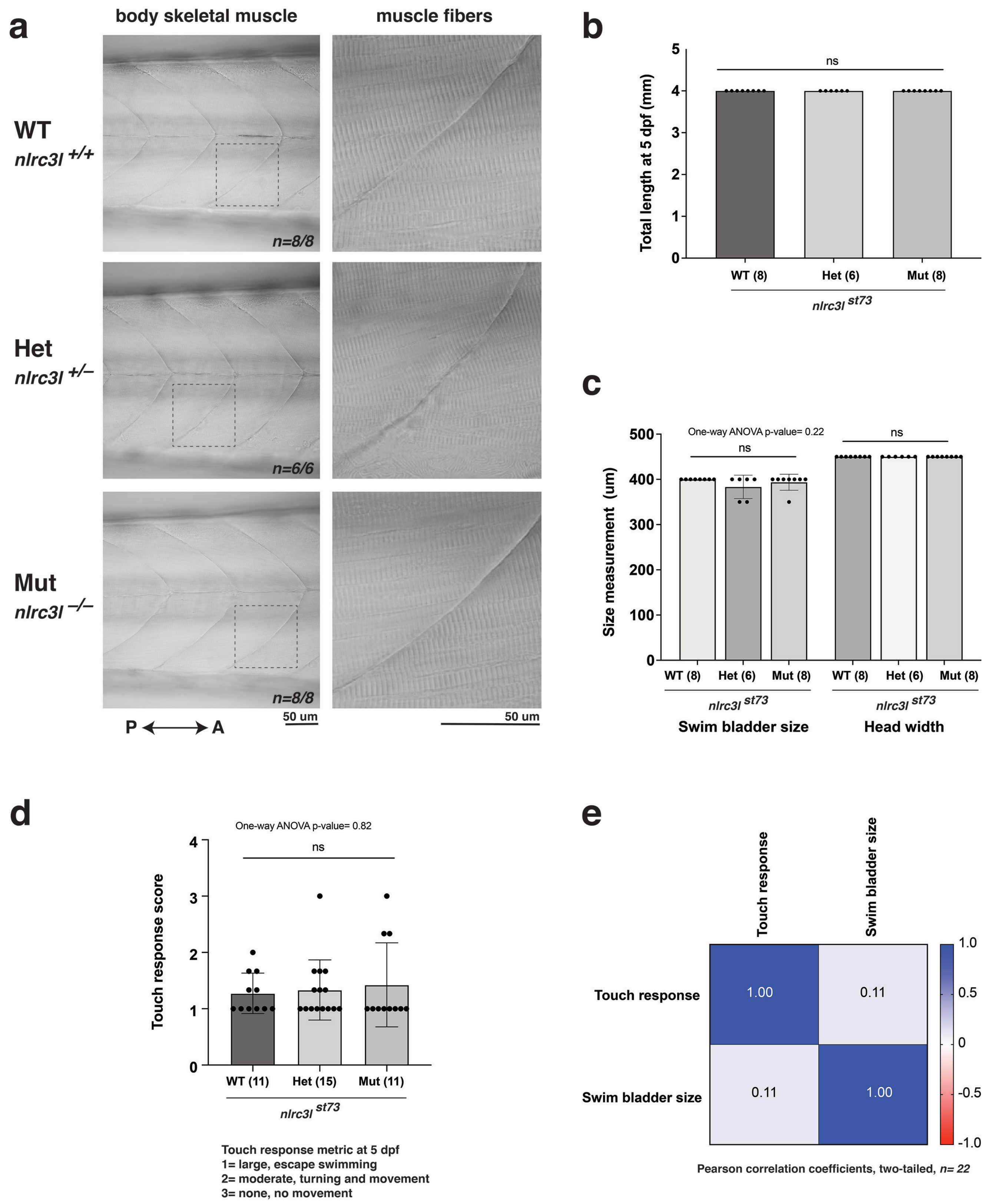

***nlrc3l* mutants have normal body sizes, skeletal muscles, and touch response indistinguishable from wild-type and heterozygous siblings at 5 dpf.**

(a) Brightfield microscopy images using a 40x objective show normal morphologies of skeletal muscles (low magnification) and fibers (high magnification in dotted box region of left panel) that are indistinguishable between *nlrc3l* mutants and control siblings. N, number of larvae analyzed. Double arrowed line shows posterior (P) and anterior (A) orientations. (b) No difference found in total length of larvae measured in mm between the genotypes. (c) No significant differences found in swim bladder size based on longest length or in head width between the genotypes. (d) Touch response assay was performed using fine forceps to gently tap trunk of larvae to evoke a stereotypical escape swimming response. Average of responses to three repeated touch tests per individual is reported. The score was based on a 3-point response metric as defined. No significant difference was found. (e) To address whether any slight variation in swim bladder sizes was associated with the touch response behavior, we conducted a Pearson correlation test on all 22 individual larvae analyzed. Results indicate no correlation between touch response to swim bladder size (Pearson coefficient= 0.11). Data supporting this figure came from two independent experiments (clutches) and all data points represent independent animals. One-way ANOVA tests were used to determine statistical significance as indicated. ns, not significant.

Supplementary Figure 2.

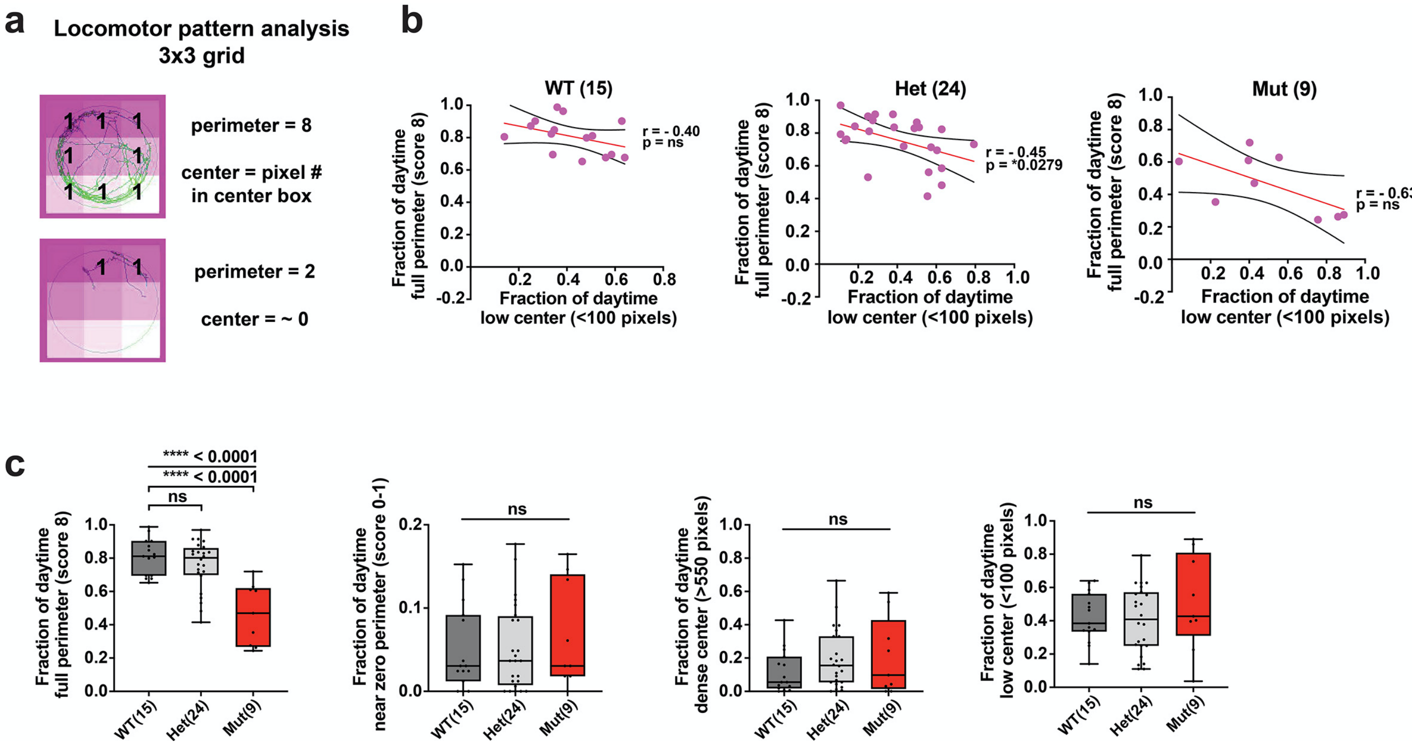

Characterization of locomotor behavior in 24-well large arenas.

(a) 3x3 grid diagram for locomotor pattern analysis. See Methods for details on analysis. (b) Plots associating maximum perimeter score with low center score for all genotypes. Pearson correlation coefficient r is shown with the associated p-value (p). (c) Scatter box-and-whisker plots show the fraction of daytime timepoints individual fish exhibit each locomotor mode (full perimeter, near zero perimeter, dense center and low center) classified by their genotype. The only significant difference shown was the reduction in full perimeter lap swimming by *nlrc3l* mutations. Each data point represents an individual animal. ns, not significant.

Supplementary Figure 3.

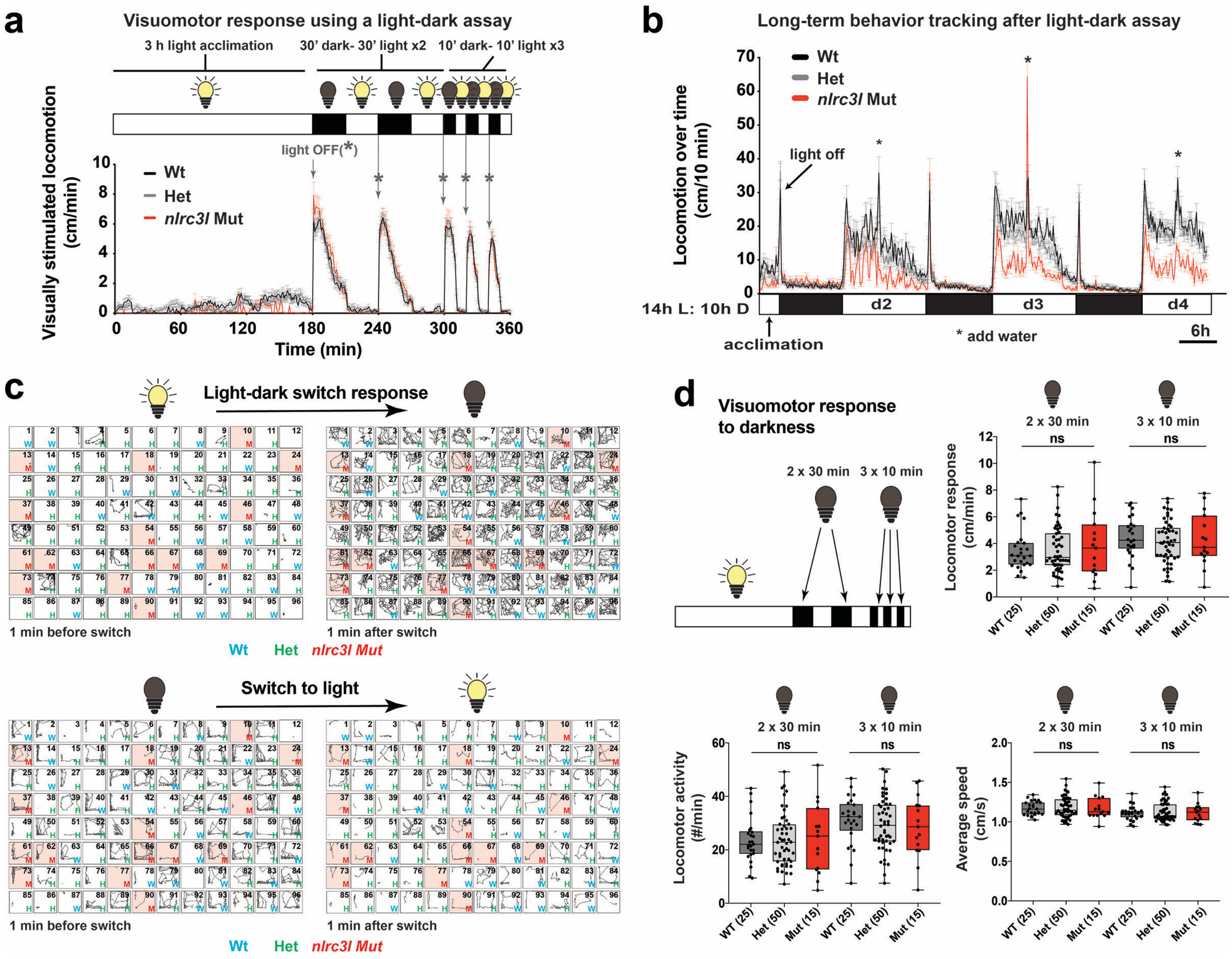

***nlrc3l* mutants show normal locomotor response to light-dark assay, but reduced daytime swimming when placed in normal day-night cycles.**

(a) Spontaneous locomotor response to visual stimuli from programmed lighting changes after a 3-hour acclimation to the light condition. Diagram at the top shows the lighting program applied in the behavior chamber where movements of 5 dpf larvae are tracked. *, light switched off event. Plots show no discrimination in behavioral response to the light-dark assay among the genotypes. Data of movements were collected in 1-minute bins. (b) Time course plot shows long-term tracking of the same larvae after the visuomotor response test in a. The expected trend in daytime

locomotion for the different genotypes over 72-hour tracking was found. Peak of movement (*) corresponds to timepoint at which each well was replenished daily with few droplets of fresh fish water. (c) Top left, movement traces at 1 minute before the light was turned off compared with traces of the same individuals (top right) at 1 minute after the light was off. A universal response to a light-to-dark switch by all fish was clear based on a rapid increase in spontaneous swimming. Bottom panels, by contrast, show no apparent locomotor response to turning on the light, although a behavioral change on a shorter timescale in seconds may be possible but not detected in our setup. Genotypes were determined after completion of the experiment and later added to the traces as shown in different color letters (W, wild-type; H, heterozygote; M, *nlrc3l* mutant; mutant wells are also shaded in light red. (d) Scatter box-and-whisker plots with error bars marking the minimum and maximum show that the locomotor response to a light-to-dark switch either in the 30-minute or 10-minute stimuli periods is not significantly different between *nlrc3l* mutants and control siblings.

Supplementary Figure 4.

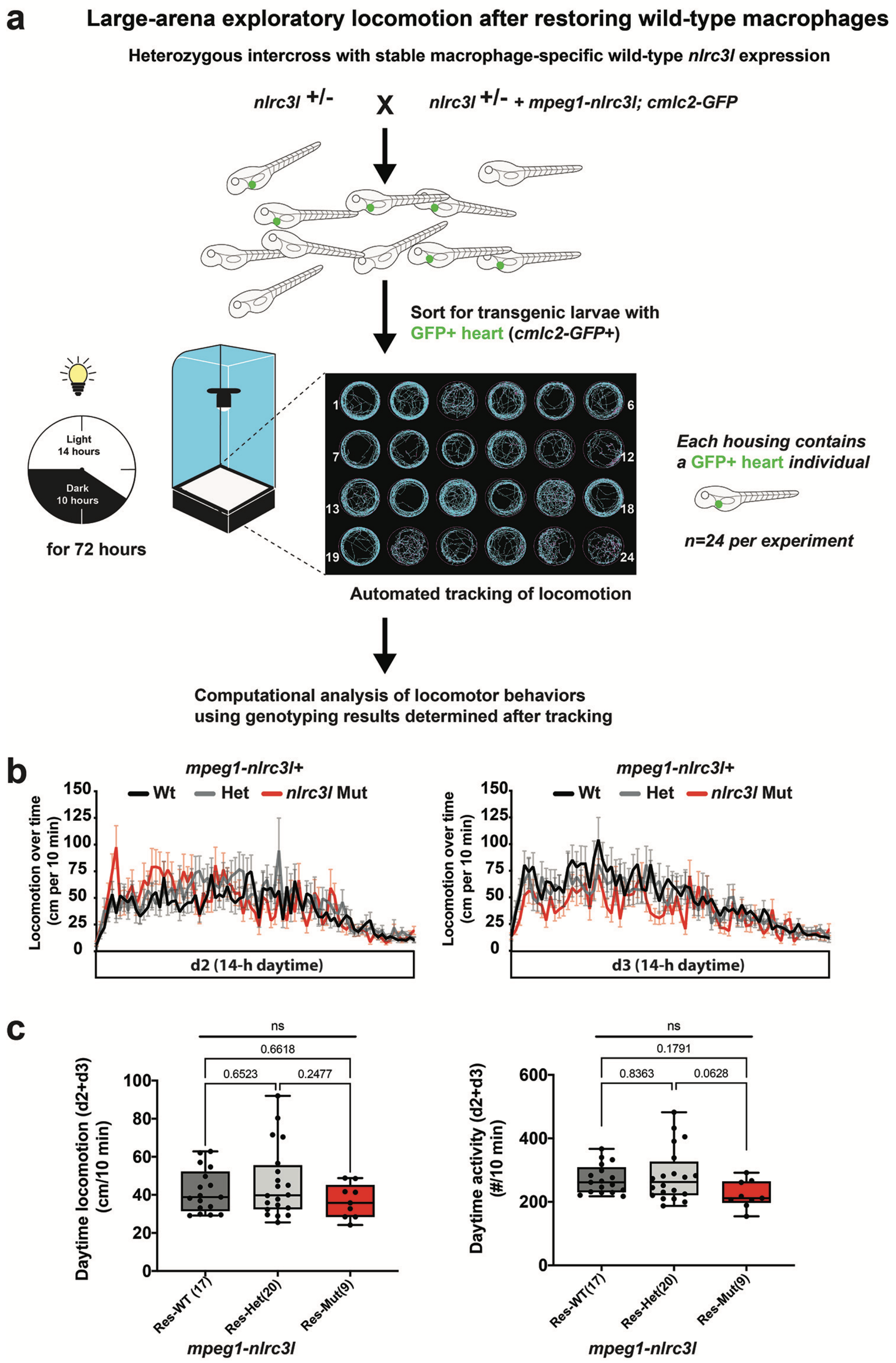

Macrophage-rescued *nlrc3l* mutants restore normal locomotor activities also in the large-arena exploratory environment.

(a) Schematic of the experimental setup for analysis of daytime locomotion after macrophage rescue in the large-arena 24-well platform. (b) Time plots showing d2 or d3 locomotion of wild-type (Wt), heterozygous (Het), and *nlrc3l* mutant (Mut) larvae with the macrophage rescue transgene (*mpeg1-nlrc3l*). Data was derived from 2 independent experiments (2 x 24-well plates). Error bars show standard error of means. (c) Scatter boxplots show no significant difference in daytime locomotion or daytime activity which were averaged over d2 and d3 for each genotype. Data indicates recovery of mutant locomotor behaviors to the typical wild-type range after macrophage rescue. Each data point represents an individual animal. ns, not significant. Significance was determined using one-way ANOVA tests followed by multiple comparisons.

Supplementary Figure 5.

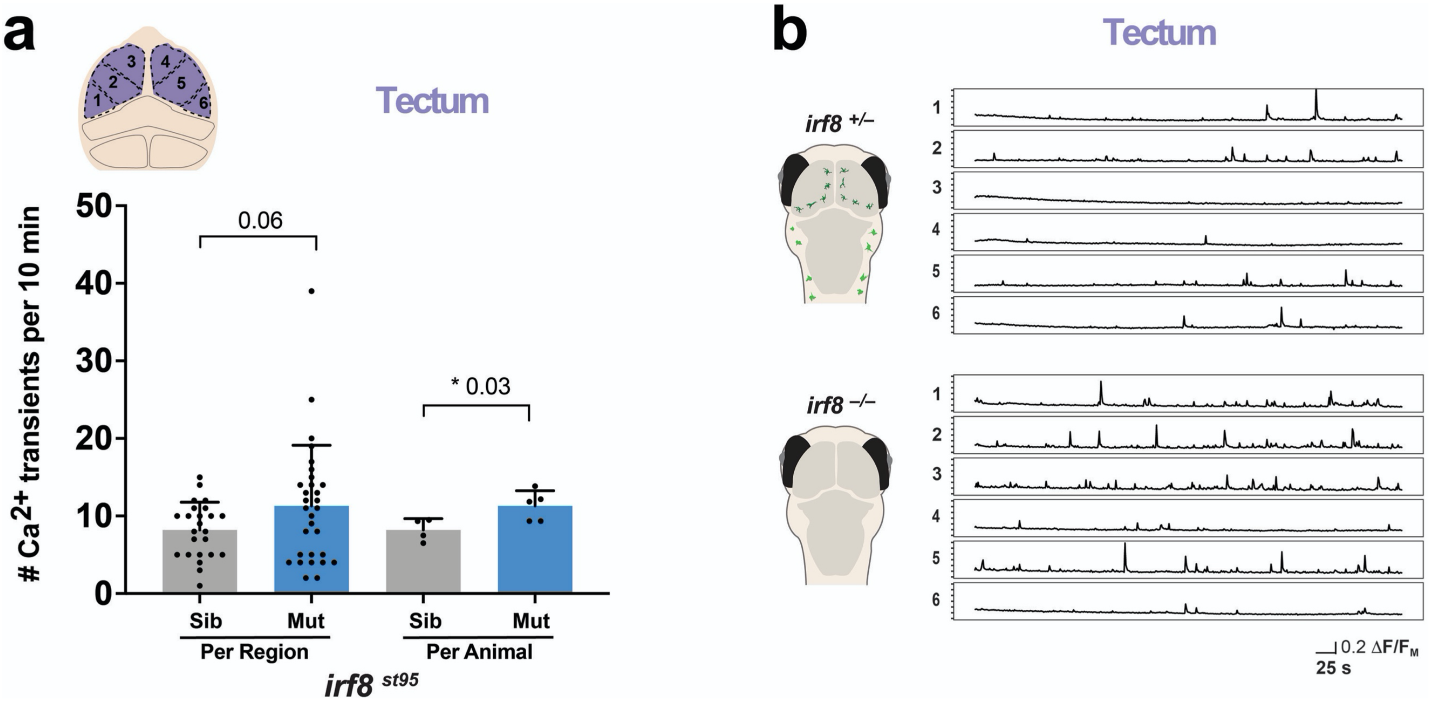

Increased tectal neuronal activity is detected in the microglia-lacking *irf8* mutants akin to *nlrc3l* mutants.

(a) *In vivo* calcium imaging shows that the number of calcium transients per tectal region trended higher in the microglia-lacking *irf8* mutant, and there is a significant increase in average number of calcium transients in the tectum per animal. Two-tailed unpaired t-test was used to determine statistical significance. (b) Representative traces of the calcium imaging over 10-minute recording show more peaks in *irf8* mutant than control sibling. Scale bar shows 0.2 change in fluorescence over mean fluorescence for y-axis, and 25 seconds scale for the x-axis.

Supplementary Figure 6.

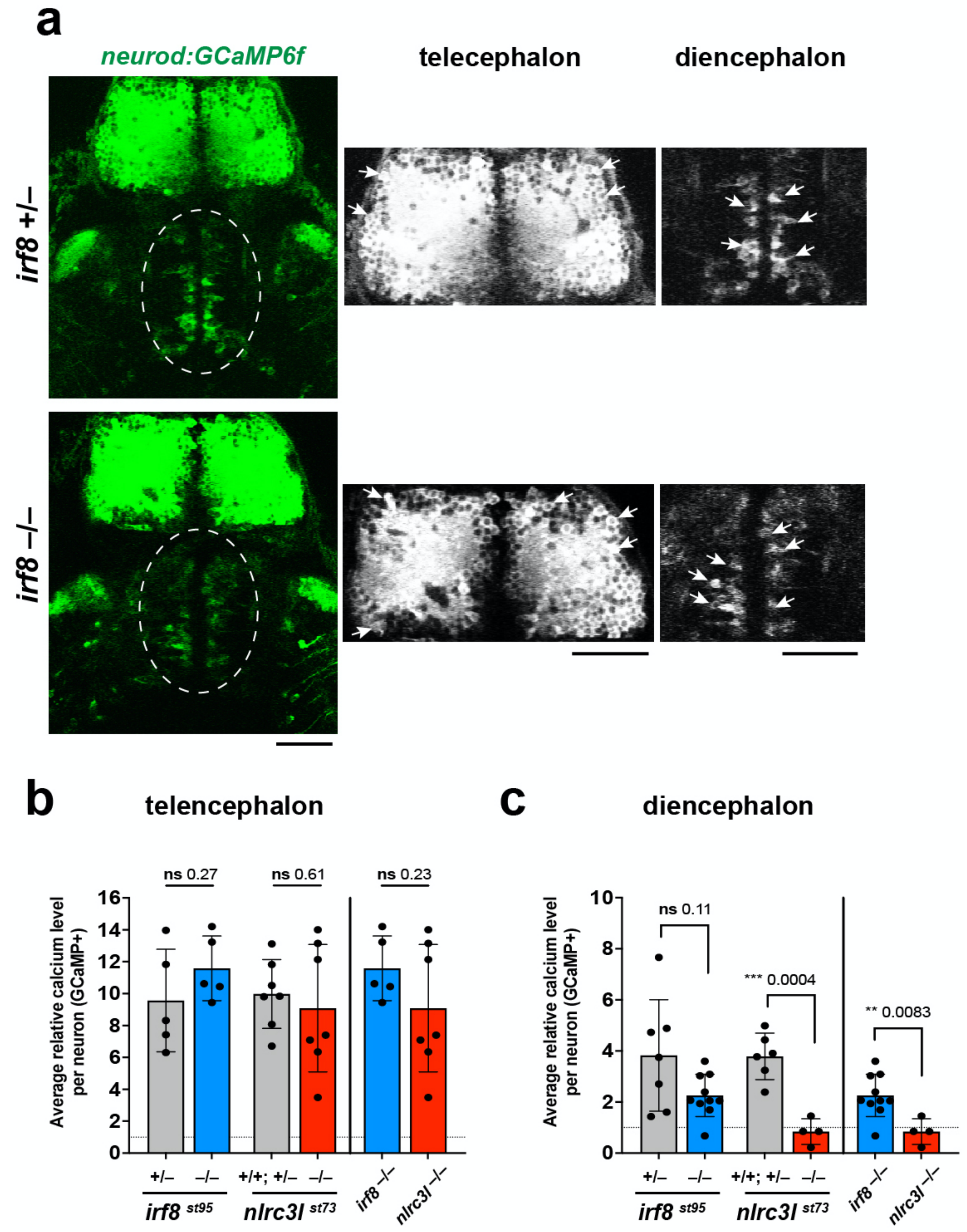

Diencephalic intracellular calcium level is not severely reduced in the microglia-lacking *irf8* mutants as it is in *nlrc3l* mutants.

(a) Representative calcium imaging of a brain slice of heterozygous sibling (top) and *irf8* mutant (bottom) showing the diencephalon (dotted circle). Right, grayscale panels show the telencephalon and diencephalon at higher magnification. Arrows, active neurons containing high levels of calcium (GCaMP6f+). Scale bars show 50 um. (b) Scatter bar chart shows no difference in intracellular calcium level in telencephalic neurons in *irf8* mutants nor in *nlrc3l* mutants. (c) Scatter bar chart shows a significant and severe reduction in *nlrc3l* mutants compared with control siblings as well as when compared with *irf8* mutants, but no significant reduction was observed in *irf8* mutants compared with control siblings. Data from main Figure 5h related to the calcium imaging of the *nlrc3l* line is plotted again here in b and c for comparison with the *irf8* cohort. Two-tailed t-tests were used to determine statistical significance. ns, not significant.

Supplementary Figure 7.

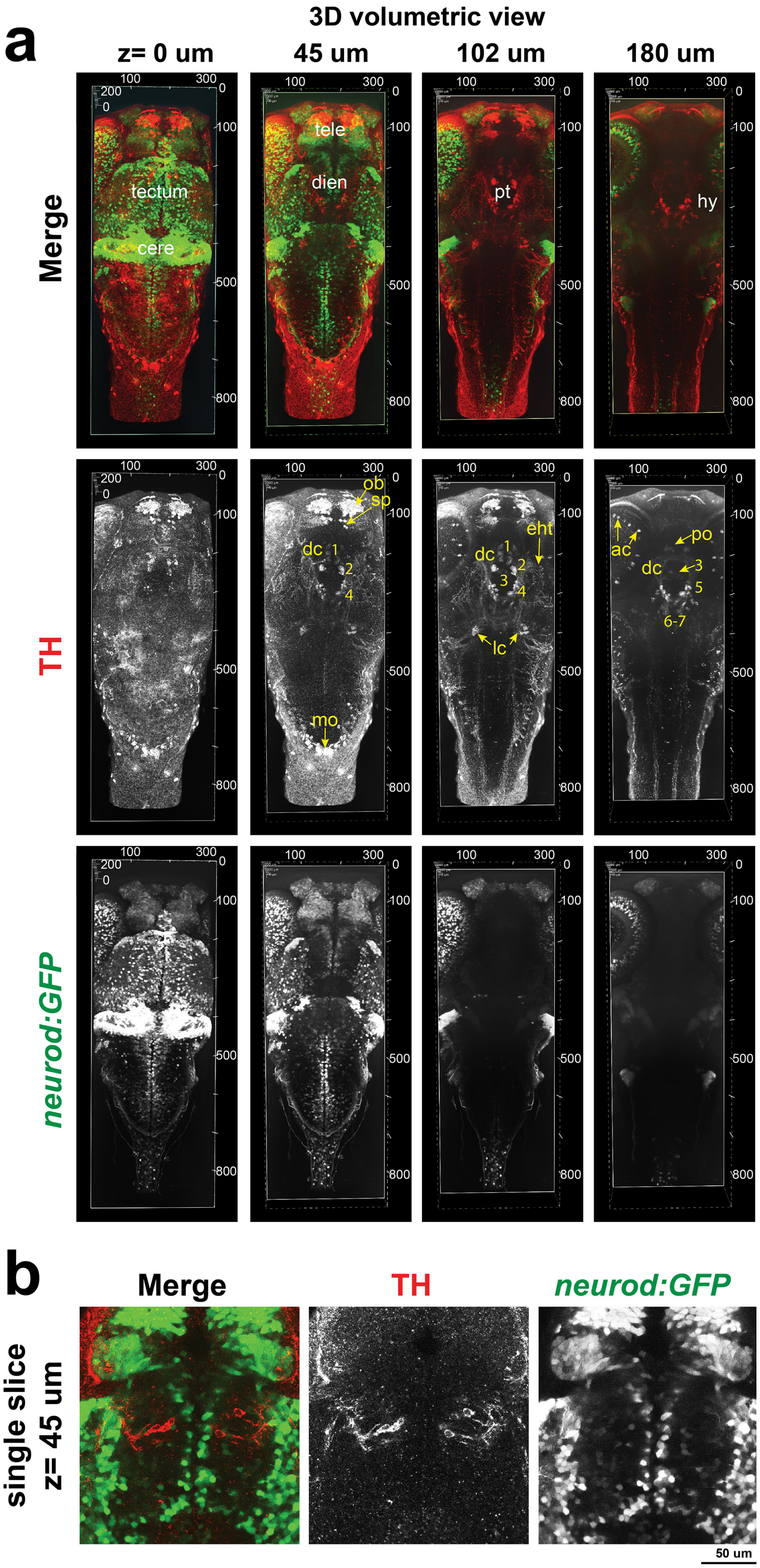

***neurod*+ neurons are different cells from the dopaminergic TH+ neurons, but they make direct contacts in the larval brain.**

(a) 3D volumetric view from 5 axial levels going from dorsal to ventral surfaces (z=0 um to z=180 um) showing TH protein expression in relation to *neurod* gene expression (*neurod:GFP*+) in wild-type 4 dpf larval zebrafish. First column at z=0 um, tectal and cerebellum neurons are strongly GFP+ but are not TH+. Second column at z= 45 um, neurons in the diencephalon corresponding to the dorsal thalamus is GFP+ and located in contact with TH+ diencephalic neurons, but they are not the same cells. Dopaminergic neurons, which are strongly TH+ in the diencephalic clusters (dc) number 1, 2, and 4, olfactory bulb (ob) and subpallium (sp)[69, 118], can be observed. Further ventrally at z= 102 um, dopaminergic neurons (TH+) can be identified in dc 1- 4; dc 2,4, and 5 make up the dopaminergic diencephalospinal neurons that directly project to the spinal circuits and provide dopamine [69]. Most ventral at z= 180 um, the dopaminergic diencephalic clusters 5-7 and remnants of dc 3 are seen. Top row, merged; middle row, red channel only (TH+); and bottom row, GFP channel only. (b) High magnification at z= 45 um showing the region corresponding to the *neurod*+ diencephalic neurons which were the subjects of calcium imaging in this study. These neurons are not TH+ but are in contact with dopaminergic neurons in the diencephalon. cere, cerebellum; tele, telencephalon; dien, diencephalon; pt, posterior tuberculum; ob, olfactory bulb; sp, subpallium; mo, medulla extrema (end of medulla oblongata); lc, locus coeruleus; ac, amacrine cells; po, postoptic region; hy, hypothalamus. Scale bar numbers are in um.

Supplementary Figure 8.

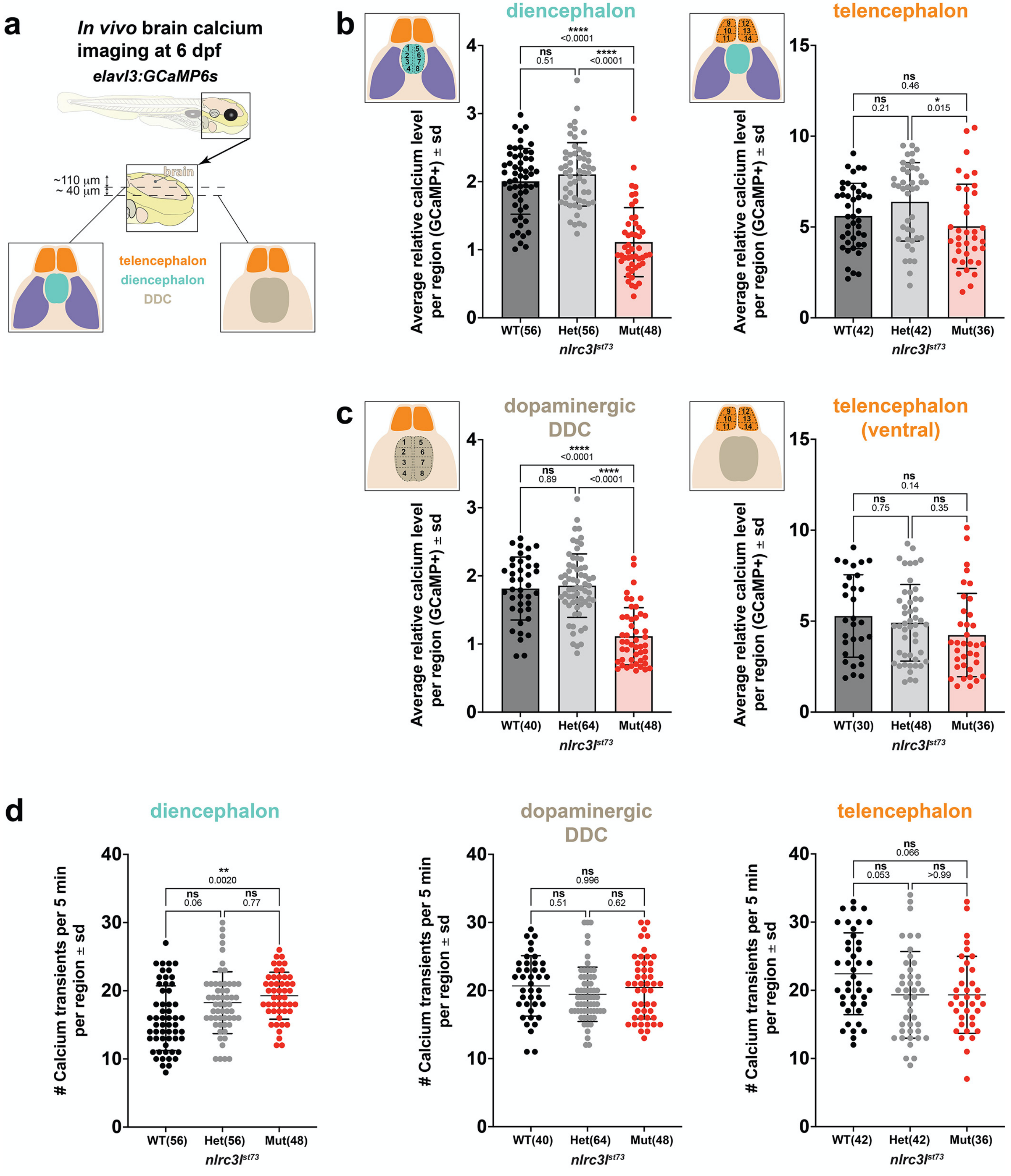

Brain pan-neuronal calcium imaging shows a significant decrease in basal intracellular calcium of diencephalic neurons in *nlrc3l* mutants, but no change in calcium transient numbers, consistent with results from targeted *neurod:GCaMP6f* imaging.

(a) Diagram illustrating the two z-planes of imaging through the diencephalon, presumptive dopaminergic diencephalic clusters (DDC), and telencephalon of the larval brain using the pan-neuronal calcium indicator *elavl3:GCaMP6s*. Here, we define the dorsal region of the diencephalon as "diencephalon" and the ventral portion as "DDC". (b-c) Scatter bar plots show a significantly large decrease in intracellular calcium level of dorsal diencephalic and DDC neurons of *nlrc3l* mutants relative to that of control siblings, but not in the telencephalon. (d) Number of calcium transients were determined over a 5-minute period. Analysis shows *nlrc3l* mutants largely had similar calcium transient numbers as their control siblings except for a possible slight increase in the dorsal diencephalic neurons compared with wild-type. Regions quantified are illustrated in the cartoons shown in b-c; diencephalon and DDC were divided into 8 relatively equal ROIs, and the telencephalon into 6 ROIs per animal analyzed. Statistical significance was determined by one-way ANOVA followed by Tukey's multiple pairwise comparisons. Corrected p-values are shown above each graph. ns, not significant. See also associated data in Supplementary Movies 6-7.

Supplementary Figure 9.

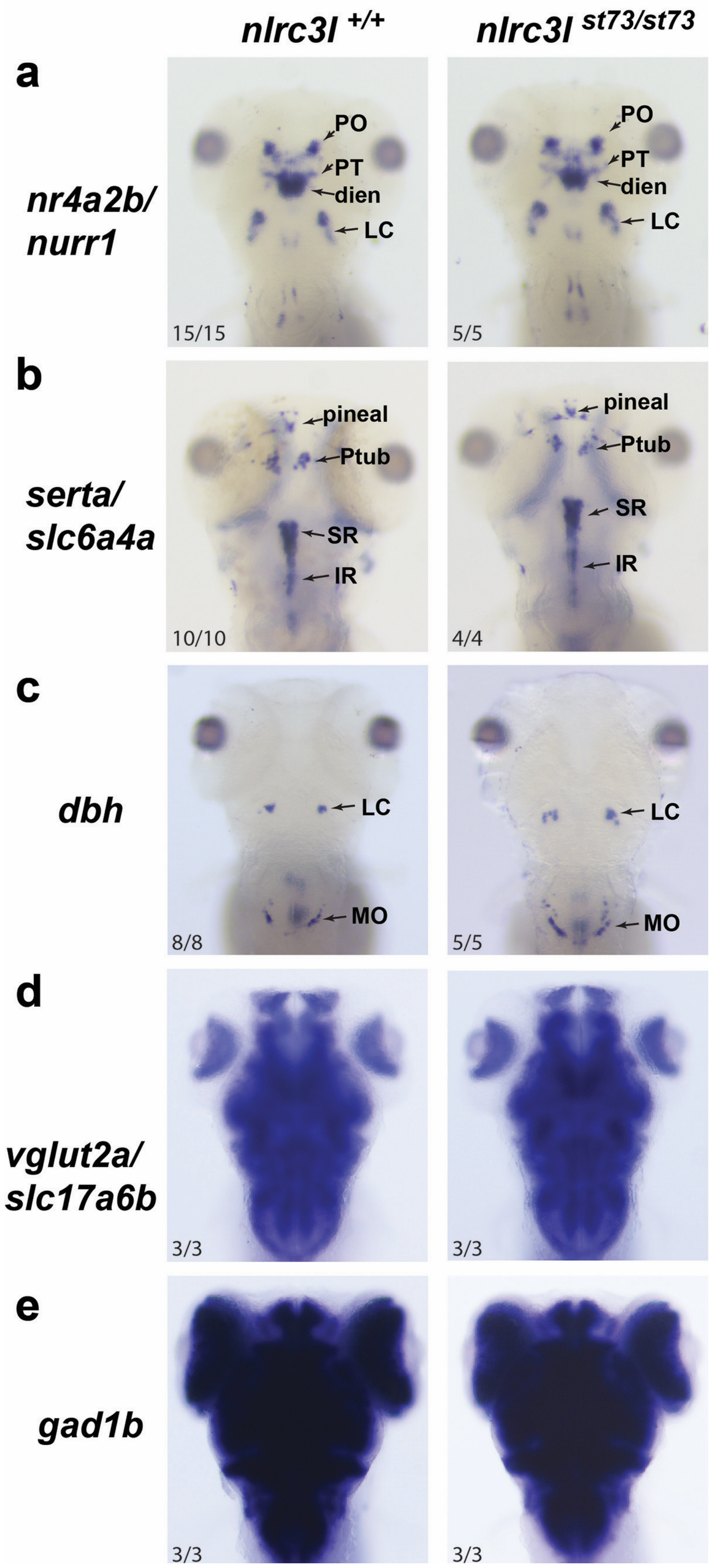

Characterization of neural structures in *nlrc3l* mutants compared with their wild-type siblings using whole mount RNA *in situ* hybridization for several neural genes.

(a) *nr4a2b* encodes for the nuclear receptor and transcription factor NURR1 that maintains the dopaminergic system and is expressed in some dopaminergic neurons[87]. Wild-type siblings and *nlrc3l* mutants do not show any apparent difference in *nr4a2b* expression (arrows). (b) *serta/slc6a4a* encodes for the serotonin transporter and its expression labels serotonergic neurons[88]; no difference was observed between the genotypes (arrows). (c) *dbh* encodes for dopamine beta-hydroxylase and its expression marks norepinephrine neurons[89]. No gross expression difference was observed between the genotypes; expression in LC ranged from discrete spot to a slightly more spread-out pattern for all genotypes. (d) *vglut2a/slc17a6b* encodes for vesicular glutamate transporter 2 and marks excitatory glutamatergic neurons[90]. Expression is broad across the CNS and no difference between genotypes was found. (e) *gad1b* encodes for glutamate decarboxylase 1 and labels inhibitory GABAergic neurons, which is broadly expressed in the CNS[90]. No apparent difference was observed between genotypes. PO, preoptic; PT, pretectum; dien, diencephalon; LC, locus coeruleus; pineal, pineal gland; Ptub, posterior tuberculum; SR, superior raphe nuclei; IR, inferior raphe nuclei; MO, medulla extrema (end of medulla oblongata). Lower left, *n*, number of animals analyzed showing similar expression pattern.

Supplementary Figure 10.

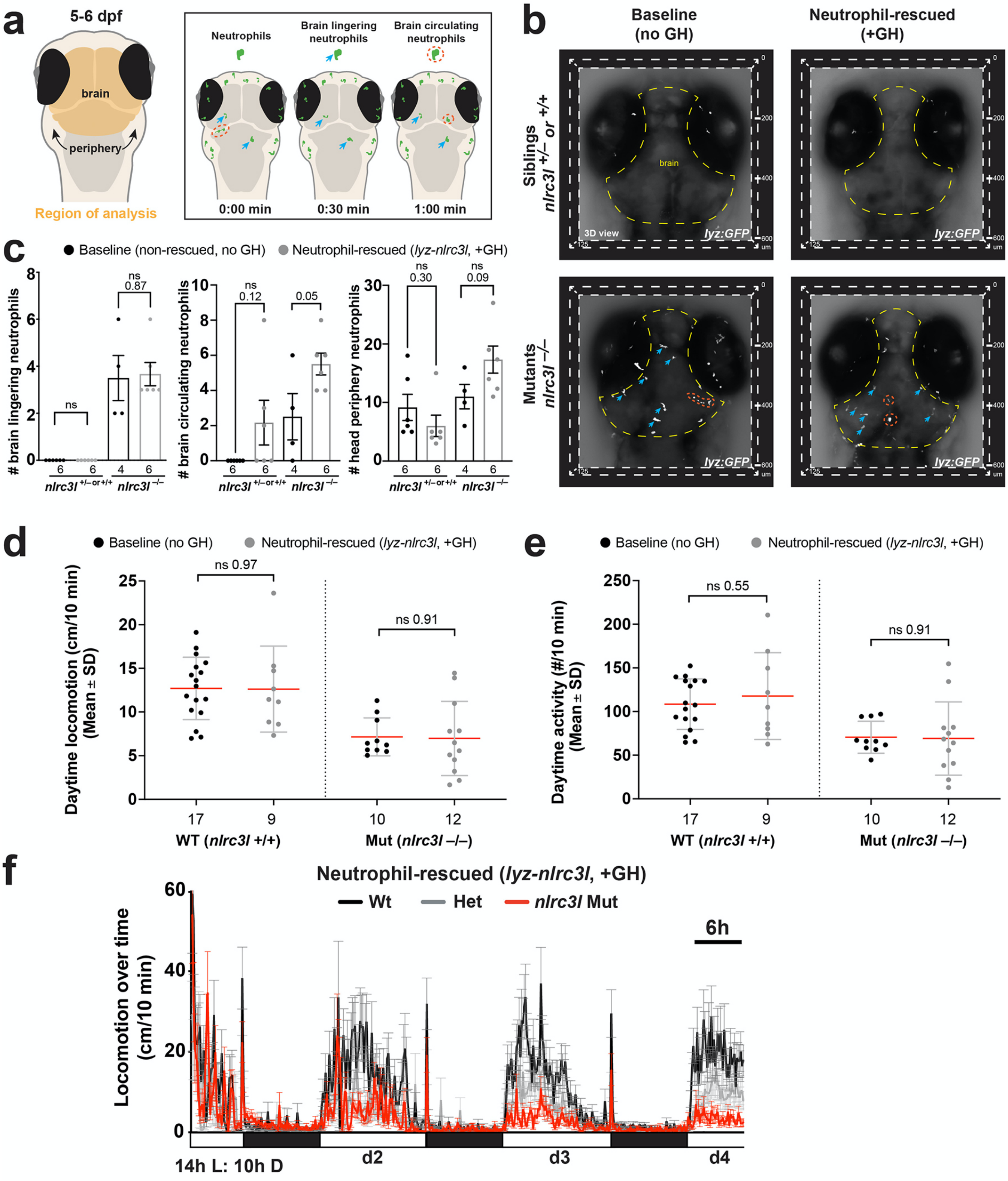

***nlrc3l* has a non-cell-autonomous role in neutrophils and their impact on locomotor behavior.**

(a) Schematic showing brain region and categories of neutrophils analyzed in 5-6 dpf larvae from *nlrc3l* heterozygous intercross with (+GH) and without (no GH) the neutrophil rescue transgene (*lyz-nlrc3l*). Brain lingering (blue arrow) defines infiltrated neutrophils that remain in the brain over the entire 5-minute period of imaging, while circulating (red dotted circle) defines neutrophils observed only at a single timepoint (imaged every 30-second) that flow through the brain. GH, GFP+ heart. (b) Representative 3D volumetric images of neutrophils (*lyz:GFP+*) in the brain at baseline and after neutrophil rescue. (c) Scatter bar plots show quantifications of brain lingering and circulating neutrophils, and total neutrophil numbers in the head periphery. Neutrophil-rescued *nlrc3l* mutants did not eliminate brain infiltration and appear phenotypically equivalent to baseline *nlrc3l* mutants. (d-f) Analysis of spontaneous locomotion over normal light:dark cycles for 72 h starting at 5 dpf of larvae derived *nlrc3l* heterozygous intercross. Two independent experiments were conducted in 96-well housings comparing individuals of different genotypes with and without the neutrophil rescue transgene side-by-side on the same dish. Genotypes were determined only after completion of experiment. Data shows that neutrophil-rescued *nlrc3l* mutants continue to show locomotor deficit in the daytime similar to the mutants at baseline. Error bars show standard error of mean. Significance was determined by two-tailed t-tests

Supplementary Figure 11.

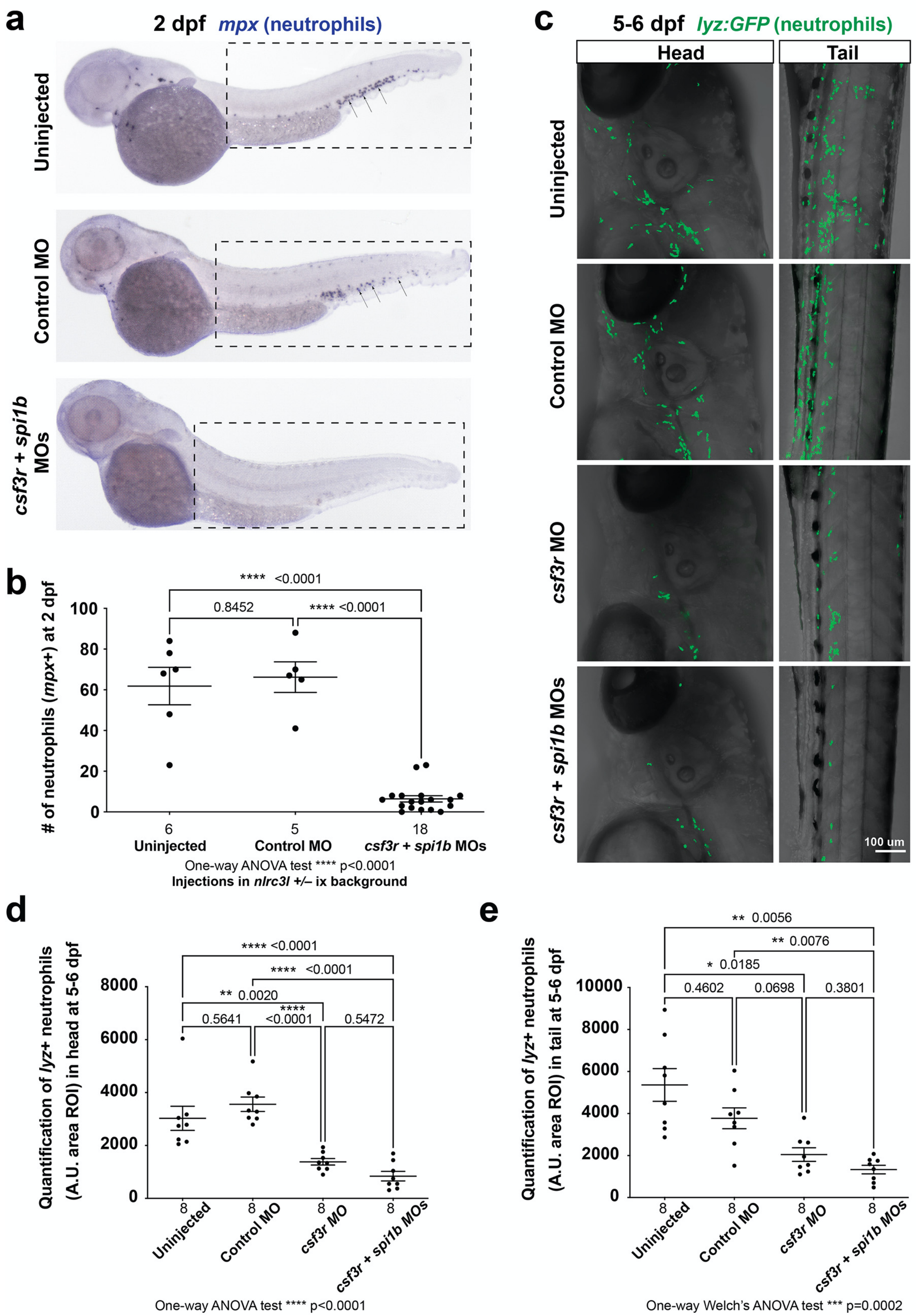

Effective depletions of neutrophils by morpholino combination against *csf3r* and *spi1b* and single morpholino against *csf3r*.

(a) Whole mount *in situ* hybridization of neutrophil-specific marker *mpx* mRNA in 2 dpf zebrafish embryos show normal distribution of neutrophils (arrows) in uninjected and control morpholino injected groups, but a severe reduction or complete ablation of neutrophils in embryos injected with a combination of *csf3r* and *spi1b* morpholinos. (b) Plot shows the number of *mpx+* neutrophils in tail region (dotted box in **a**) was reduced on average by 90% in *csf3r/spi1b* MOs-injected animals compared to uninjected and control morpholino-injected individuals. The efficacy of the double morpholino treatment for neutrophil ablation in *nlrc3l* heterozygous incross background was high and showed no difference between genotypes. (c) Injections of *csf3r/spi1b* combined morpholinos and *csf3r* morpholino alone in wild-type embryos showed high efficacy in depleting neutrophils even at later larval stages at 5-6 dpf as shown by confocal imaging of neutrophils using the *lyz:GFP* reporter. Quantifications of the (d) head region, and (e) tail region show significant depletion of neutrophils by combined *csf3r/spi1b* MOs or single *csf3r* MO as compared to uninjected and control MO groups. Numbers below bar graphs represent *n*, number of embryos analyzed. Error bars show standard error of means. Statistical significance was determined by one-way ANOVA, or one-way Welch's ANOVA given significant differences in standard deviations, followed by multiple comparison tests; MO, morpholino.
