# Supplementary material for: Peripheral NOD-like receptor deficient inflammatory macrophages trigger neutrophil infiltration into the brain disrupting daytime locomotion
Source: Commun Biol. 2022 May 16;5:464. doi: 10.1038/s42003-022-03410-z (PMC9110401; doi:10.1038/s42003-022-03410-z)
Supplement: Supplementary file 2 — Description of Additional Supplementary Files [file 42003_2022_3410_MOESM2_ESM.pdf]

Description of Additional Supplementary Files

File name: Supplementary Movie 1
Description: Short clip of larval zebrafish movements in 96-well format. A representative video tracking of a partial 96-well plate showing 54 individual larvae (9 x 6 square wells) during the daytime inside the ZebraBox. Zebrafish show varied behaviors from active bouts of swimming to minimal or no movement at any given timepoint as shown in this short 2-minute tracking. Movie is shown at 4X playback speed.

File name: Supplementary Movie 2
Description: Short clip of larval zebrafish daytime movements in 24-well. Movie shows a short clip of behavioral tracking of 24 individual zebrafish larvae in the daytime (44 seconds in real time). Fish were derived from a nlrc3lst73 heterozygous intercross. Each well is annotated by the corresponding genotype of the residing fish as determined after completion of data collection. Traces indicate a range of movements from rapid bouts of perimeter swimming to slow or no movement in all genotypes, conveying the natural variation in spontaneous locomotion independent of genotype at any given time.

File name: Supplementary Movie 3
Description: Traces from simultaneous tracking of larval zebrafish in 24-well platform show inter-and intra-individual variation in spontaneous locomotion over three normal day-night cycles. Movie shows swimming traces of individual larvaes (red trajectories in each circular well) in chronological order at 30 fps. 24 zebrafish were simultaneously and continuously tracked for 72 hours under the normal 14-hour light: 10-hour dark cycles. Each frame is a 10-minute integration of movements. The larval zebrafish were derived from a nlrc3lst73 heterozygous intercross, monitored starting at 5 dpf in individual circular wells, and genotyped after tracking. Fish genotype is annotated on each well during the 14- hour daytime periods, and timepoint is displayed on the lower right corner of each plate to account for a total of 432 timepoints, each representing a 10-minute integration and in sum covering a 72-hour duration.

File name: Supplementary Movie 4
Description: Microglia are distributed throughout larval zebrafish brain and not exclusive to the tectum. Movie shows a 3D rendering of 5 dpf wild-type whole brain in vivo from a large multi-tiled confocal z-stack taken at 40x using 1um z-slices. mpeg1:GFP transgene was used to visualize microglia. The z-stack is about 250 um in z-depth, 320 um along the x-axis, and 600 um along the y-axis. The transgenic 23 expression of mpeg1:GFP is depth-coded from magenta at z= 0 um in the most ventral surface to cyan at z= 250 um at the most dorsal surface. The most superficial expression of the transgene as shown in shades of blue surrounding the larval brain appears to be skin autofluorescence, and can be used as an anatomical reference. Individual cells labeled by mpeg1:GFP beneath the autofluorescent exterior layer are microglia, which appear morphologically diverse in shape and size (some ramified with long process extensions while others are rounded). The movie scrolls through the volume from dorsal to ventral and back out to dorsal surface, then makes a 90-degree turn to show the transverse view of the volume from posterior to anterior. See corresponding data in Figure 4a.

File name: Supplementary Movie 5
Description: Microglia closely intermix with neurons throughout the larval zebrafish brain. Movie shows a 3D rendering of 5 dpf wild-type whole brain in vivo using a single tile confocal z-stack taken

at 40x from 1um z-slices. mpeg1:GFP and nbt:dsRed transgenes were used to label microglia and neurons, respectively. The z-stack is about 325 um in z-depth, 320 um along the x-axis, and 320 um along the y-axis. Microglia cells (green) are clearly embedded between and among neurons (red) both in the dorsal and ventral regions of the brain. The skin shows autofluorescence from mpeg1:GFP and can be used as an anatomical reference. The movie scrolls through the volume from dorsal to ventral and back out to dorsal surface, then makes a 90-degree turn to show the transverse view of the volume from posterior to anterior. See corresponding data in Figure 4b.

File name: Supplementary Movie 6
Description: In vivo brain calcium imaging reveals spontaneous neural activity and calcium levels in the larval nlrc3l mutant. Movie shows time-lapse imaging of an optical z-slice through the telencephalon (tele) and diencephalon (dien) in the 6 dpf nlrc3l mutant zebrafish larva carrying the pan-neuronal calcium indicator elavl3:GCaMP6s. 40x objective was used to capture images at 1 Hz. File shows 30 fps.

File name: Supplementary Movie 7
Description: In vivo brain calcium imaging reveals spontaneous neural activity and calcium levels in the larval wild-type sibling. Movie shows time-lapse imaging of an optical z-slice through the telencephalon (tele) and diencephalon (dien) in the 6 dpf wild-type zebrafish larva carrying the pan-neuronal calcium indicator elavl3:GCaMP6s. 40x objective was used to capture images at 1 Hz. File shows 30 fps.

File name: Supplementary Movie 8
Description: Neutrophils circulate through and infiltrate the diencephalon of the larval nlrc3l mutant at 6 dpf. In vivo time-lapse imaging of 6 dpf double transgenic nlrc3l mutant brain carrying the neutrophil reporter lyz:GFP and pan-neuronal reporter nbt:dsRed using a 40x objective captured at 1 scan every 30 seconds for 40 minutes. Movie shows maximum projection of 25 slices encompassing a 50-um z-stack corresponding to region between tectum and ventral diencephalon at 30 fps. Top left corner shows actual time stamp in minutes:seconds. Neutrophils are prominently observed passing through and infiltrating the diencephalon.

File name: Supplementary Movie 9
Description: Baseline nlrc3l mutants show abundant neutrophil activity in the larval brain. Representative in vivo time-lapse confocal imaging of brain from 5 dpf transgenic nlrc3l mutant carrying the neutrophil reporter lyz:GFP was conducted using a 20x objective captured at 1 scan every 30 seconds for 15 minutes. Movie shows a maximum projection of 25 slices encompassing a 125-um z-stack. Bottom right corner shows actual time stamp in minutes:seconds. Arrows point to "brain lingering" neutrophils surveying the brain and "circulating" neutrophils with blood flow. Movie shows 4 fps. See corresponding analysis in Figure 7.

File name: Supplementary Movie 10
Description: Baseline control siblings show the expected lack of neutrophil activity in larval brain. Representative in vivo time-lapse imaging of brain from 5 dpf transgenic nlrc3l heterozygous sibling carrying the neutrophil reporter lyz:GFP was taken using a 20x objective captured at 1 scan every 30 seconds for 15 minutes. Movie shows a maximum projection of 25 slices encompassing a 125-um z-stack. Bottom right corner shows actual time stamp in minutes:seconds. No neutrophil infiltration was observed in the brain. Movie shows 4 fps. See corresponding analysis in Figure 7.

File name: Supplementary Movie 11
Description: Infiltration of neutrophils in the nlrc3l mutant brain begins as early as 2 dpf. In vivo

time-lapse imaging of brain from 2 dpf transgenic nlrc3l mutant carrying the neutrophil reporter lyz:GFP and pan-neuronal reporter nbt:dsRed using a 40x objective captured at 1 scan every 30 seconds for ~40 minutes. Movie shows maximum projection of 21 slices encompassing a 42-um z-stack corresponding to the transverse view of the tectal midbrain, hindbrain, and 25 ventricle at 30 fps. Top left corner shows actual time stamp in minutes:seconds. Neutrophils are abnormally circulating through the brain and several have resided in the brain dynamically moving about in the hindbrain and ventral brain.

File name: Supplementary Movie 12
Description: Macrophage-rescued nlrc3l mutants show a reversal of neutrophil infiltration in the larval brain. Representative in vivo time-lapse confocal imaging of brain from 5 dpf transgenic nlrc3l mutant carrying the macrophage-rescue cassette (mpeg1:nlrc3l) and neutrophil reporter lyz:GFP was conducted using a 20x objective captured at 1 scan every 30 seconds for 15 minutes. Movie shows a maximum projection of 25 slices encompassing a 125-um z-stack. Bottom right corner shows actual time stamp in minutes:seconds. No neutrophils were found lingering in the brain and few circulating neutrophils were observed after restoring wild-type nlrc3l expression specifically in macrophages. Movie shows 4 fps. See corresponding analysis in Figure 7.

File name: Supplementary Movie 13
Description: Macrophage-specific rescue construct has no apparent effect on control siblings. Representative in vivo time-lapse imaging of brain from 5 dpf transgenic wild-type sibling carrying the macrophage-rescue cassette (mpeg1:nlrc3l) and neutrophil reporter lyz:GFP was taken using a 20x objective captured at 1 scan every 30 seconds for 15 minutes. Movie shows a maximum projection of 25 slices encompassing a 125-um z-stack. Bottom right corner shows actual time stamp in minutes:seconds. Movie shows 4 fps. No neutrophil infiltration was observed in the brain. See corresponding analysis in Figure 7.

File name: Supplementary Data 1
Description: Source data.
