# Supplementary material for: Peripheral NOD-like receptor deficient inflammatory macrophages trigger neutrophil infiltration into the brain disrupting daytime locomotion
Source: Commun Biol. 2022 May 16;5:464. doi: 10.1038/s42003-022-03410-z (PMC9110401; doi:10.1038/s42003-022-03410-z)
Supplement: Supplementary file 17 — Reporting Summary [file 42003_2022_3410_MOESM17_ESM.pdf]

nature portfolio

Corresponding author(s): Celia Shiau

Last updated by author(s): 4/13/2022

Reporting Summary

Nature Portfolio wishes to improve the reproducibility of the work that we publish. This form provides structure for consistency and transparency in reporting. For further information on Nature Portfolio policies, see our Editorial Policies and the Editorial Policy Checklist.

Statistics

For all statistical analyses, confirm that the following items are present in the figure legend, table legend, main text, or Methods section.

n/a	Confirmed	
☐	☒	The exact sample size (*n*) for each experimental group/condition, given as a discrete number and unit of measurement
☐	☒	A statement on whether measurements were taken from distinct samples or whether the same sample was measured repeatedly
☐	☒	The statistical test(s) used AND whether they are one- or two-sided<br>*Only common tests should be described solely by name; describe more complex techniques in the Methods section.*
☐	☒	A description of all covariates tested
☐	☒	A description of any assumptions or corrections, such as tests of normality and adjustment for multiple comparisons
☐	☒	A full description of the statistical parameters including central tendency (e.g. means) or other basic estimates (e.g. regression coefficient) AND variation (e.g. standard deviation) or associated estimates of uncertainty (e.g. confidence intervals)
☐	☒	For null hypothesis testing, the test statistic (e.g. *F*, *t*, *r*) with confidence intervals, effect sizes, degrees of freedom and *P* value noted<br>*Give P values as exact values whenever suitable.*
☒	☐	For Bayesian analysis, information on the choice of priors and Markov chain Monte Carlo settings
☒	☐	For hierarchical and complex designs, identification of the appropriate level for tests and full reporting of outcomes
☒	☐	Estimates of effect sizes (e.g. Cohen's *d*, Pearson's *r*), indicating how they were calculated

Our web collection on statistics for biologists contains articles on many of the points above.

Software and code

Policy information about availability of computer code

Data collection	*Provide a description of all commercial, open source and custom code used to collect the data in this study, specifying the version used OR state that no software was used.*
Data analysis	*Provide a description of all commercial, open source and custom code used to analyse the data in this study, specifying the version used OR state that no software was used.*

For manuscripts utilizing custom algorithms or software that are central to the research but not yet described in published literature, software must be made available to editors and reviewers. We strongly encourage code deposition in a community repository (e.g. GitHub). See the Nature Portfolio guidelines for submitting code & software for further information.

Data

Policy information about availability of data

All manuscripts must include a data availability statement. This statement should provide the following information, where applicable:

- Accession codes, unique identifiers, or web links for publicly available datasets
- A description of any restrictions on data availability
- For clinical datasets or third party data, please ensure that the statement adheres to our policy

All data generated or analyzed during this study are included in this published article (and its supplementary information files). Any additional files related to the datasets in the current study are available from the corresponding author on reasonable request.

Field-specific reporting

Please select the one below that is the best fit for your research. If you are not sure, read the appropriate sections before making your selection.

☒ Life sciences ☐ Behavioural & social sciences ☐ Ecological, evolutionary & environmental sciences

For a reference copy of the document with all sections, see nature.com/documents/nr-reporting-summary-flat.pdf

Life sciences study design

All studies must disclose on these points even when the disclosure is negative.

Sample size	Sample size was determined as the number of biological replicates larger than the minimum sample size sufficient to detect significant differences between two or more groups by power analysis. Experiments were designed for sample size of 4-6 or more, and as large as > 50.
Data exclusions	No exclusions unless there were technical errors where the experiment could not be used.
Replication	All experiments were repeated at least twice, and biological replicates are used for each N for sample size.
Randomization	All samples were randomized at time of collection, and genotypes only determined and associated for analysis of data after completion of experiment.
Blinding	Genotypes were blinded to researcher at time of experimentation for all experiments.

Reporting for specific materials, systems and methods

We require information from authors about some types of materials, experimental systems and methods used in many studies. Here, indicate whether each material, system or method listed is relevant to your study. If you are not sure if a list item applies to your research, read the appropriate section before selecting a response.

Materials & experimental systems

n/a	Involved in the study	
☐	☒	Antibodies
☒	☐	Eukaryotic cell lines
☒	☐	Palaeontology and archaeology
☐	☒	Animals and other organisms
☒	☐	Human research participants
☒	☐	Clinical data
☒	☐	Dual use research of concern

Methods

n/a	Involved in the study	
☒	☐	ChIP-seq
☒	☐	Flow cytometry
☒	☐	MRI-based neuroimaging

Antibodies

Antibodies used	Primary rabbit anti-Tyrosine Hydroxylase antibody (AB152, Sigma Aldrich) and secondary goat anti-rabbit Alexa Fluor 594 were used. (ThermoFisher).
Validation	Validation based on known IHC staining published previously as cited in the paper.

Animals and other organisms

Policy information about studies involving animals; ARRIVE guidelines recommended for reporting animal research

Laboratory animals	Embryos from wild-type (TL and AB), mutant and transgenic backgrounds: nlrc3lst73 28, irf8st95 48, xpr1bst89 49, pu.1/spi1bfh509 50, mpeg1:EGFP 112, lyz:GFP 113, neurod:GCaMP6f 75, and nbt:DsRed 114 were raised at 28.5°C and staged as described115. Stable transgenic mpeg1:nlrc3l and lyz:nlrc3l lines were generated using Tol2-mediated transgenesis of constructs made from integrating full-length wild-type nlrc3l coding sequence downstream of regulatory sequence for mpeg1(1.86 kb) or lyz(2.4 kb), respectively, using gateway plasmids previously created28; these constructs were validated by Sanger sequencing. Stable elavl3:GCaMP6s line was generated using Tol2-mediated transgenesis. This study was carried out in accordance with the approval of UNC-Chapel Hill Institutional Animal Care and Use Committee (protocols 16–160 and 19–132).
Wild animals	*Provide details on animals observed in or captured in the field; report species, sex and age where possible. Describe how animals were caught and transported and what happened to captive animals after the study (if killed, explain why and describe method; if released, say where and when) OR state that the study did not involve wild animals.*

Field-collected samples	*For laboratory work with field-collected samples, describe all relevant parameters such as housing, maintenance, temperature, photoperiod and end-of-experiment protocol OR state that the study did not involve samples collected from the field.*
Ethics oversight	*Identify the organization(s) that approved or provided guidance on the study protocol, OR state that no ethical approval or guidance was required and explain why not.*

Note that full information on the approval of the study protocol must also be provided in the manuscript.
